# Antimicrobial Properties of Diamond-Like Carbon/Silver Nanocomposite Thin Films Deposited on Textiles: Towards Smart Bandages

**DOI:** 10.3390/ma9050371

**Published:** 2016-05-13

**Authors:** Tadas Juknius, Modestas Ružauskas, Tomas Tamulevičius, Rita Šiugždinienė, Indrė Juknienė, Andrius Vasiliauskas, Aušrinė Jurkevičiūtė, Sigitas Tamulevičius

**Affiliations:** 1 Institute of Materials Science, Kaunas University of Technology, K. Baršausko St. 59, 51423 Kaunas, Lithuania; Tadas.Juknius@ktu.edu (T.J.); Ausrine.Jurkeviciute@ktu.lt (A.J.); Sigitas.Tamulevicius@ktu.lt (S.T.); 2 Veterinary Academy, Lithuanian University of Health Sciences, Tilžės St. 18, 47181 Kaunas, Lithuania; Modestas.Ruzauskas@lsmuni.lt (M.R.); Rita.Siugzdiniene@lsmuni.lt (R.Š.); Indre.Jukniene@fc.lsmuni.lt (I.J.); Andrius.Vasiliauskas@ktu.lt (A.V.); 3 Department of Physics, Kaunas University of Technology, Studentų St. 50, 51368 Kaunas, Lithuania

**Keywords:** nanocomposite, silver, bandage, antimicrobial, *S. aureus*, 81.07.-b, AGRICOLASCC: L832

## Abstract

In the current work, a new antibacterial bandage was proposed where diamond-like carbon with silver nanoparticle (DLC:Ag)-coated synthetic silk tissue was used as a building block. The DLC:Ag structure, the dimensions of nanoparticles, the silver concentration and the silver ion release were studied systematically employing scanning electron microscopy, energy dispersive X-ray spectroscopy and atomic absorption spectroscopy, respectively. Antimicrobial properties were investigated using microbiological tests (disk diffusion method and spread-plate technique). The DLC:Ag layer was stabilized on the surface of the bandage using a thin layer of medical grade gelatin and cellulose. Four different strains of *Staphylococcus aureus* extracted from humans’ and animals’ infected wounds were used. It is demonstrated that the efficiency of the Ag^+^ ion release to the aqueous media can be increased by further RF oxygen plasma etching of the nanocomposite. It was obtained that the best antibacterial properties were demonstrated by the plasma-processed DLC:Ag layer having a 3.12 at % Ag surface concentration with the dominating linear dimensions of nanoparticles being 23.7 nm. An extra protective layer made from cellulose and gelatin with agar contributed to the accumulation and efficient release of silver ions to the aqueous media, increasing bandage antimicrobial efficiency up to 50% as compared to the single DLC:Ag layer on textile.

## 1. Introduction

Quite often, due to bad care of a wound (or a weak immune system), pathogenic microorganisms cause wound inflammation. Bacteria, like methicillin-resistant *Staphylococcus aureus* (MRSA), can cause severe infections with bad prognosis and consequences [1,2,3]. On the other hand, small wounds after injuries can be healed using simple cotton bandages, which usually do not protect the damaged tissues from bacteria and secondary infection.

It is known that bacterial adhesion is the first step in colonization of wounded skin and the formation of a biofilm [4]. Therefore, it is very important to have the antibacterial surface directly on the bandage to avoid the formation of these bacteria films, preventing bacteria multiplication inside the bandage [5]. Bacterial infections are usually treated with antibiotics; however, a good effect is not obtained in every case, and this is because many bacteria are immunized to antibiotics after being subjected to the treatment [6]. Moreover, antimicrobial resistance is directly associated with over-usage of antibiotics; thus, alternative methods for treatment are necessary. One of the most important pathogenic bacteria associated with different types of infections, including skin and soft tissues, is *S. aureus* [7]. Infections caused by this species sometimes are very hard to treat because of its multiple-resistance. Due to this reason, *S. aureus* is reputed as one of the most intractable pathogenic bacteria in the history of antibiotic chemotherapy [8].

The closure of wounds and fistulas using silver sutures was shown to be very successful in preventing infections [9]. Silver can be applied in different forms, namely as a metal, as a compound or as a free dissolved ion. The famous Hippocrates first described silver’s antimicrobial properties in 400 BC [10]. The ancient users of silver had no idea what form of silver worked best, but they just observed the positive effects of silver and silver salts. They also realized that silver worked best when some moisture was present. Now, it is proven that the silver ions (Ag^+^) are responsible for the antimicrobial activity [11]. It is known that silver is an antiseptic metal and can act against bacteria in different ways due to Ag cation release in aqueous media. Ag^+^ can destruct the cell membranes, destroy the respiratory enzyme system or can block DNA replication. This variability of antibacterial mechanisms of Ag hinders bacterial resistance formation [12]. The possible antibacterial action mechanisms of silver nanoparticles (Ag NPs) are explained in the following way. Ag^+^ ions are supposed to bind to sulfhydryl groups, which lead to protein denaturation by reducing disulfide bonds; Ag^+^ can complex with electron donor groups containing sulfur, oxygen or nitrogen that are normally present as thiols or phosphates on amino acids and nucleic acids [13]. Ag NPs react with sulfur-rich proteins in the bacteria cell membrane and the interior of the cell or with phosphorous-containing compounds, such as DNA. Accordingly, the morphological changes in the bacteria cell membrane and the possible damage of DNA caused by the reaction with Ag NPs disturb the respiratory chain or cell division processes, leading to cell death [14]. The antibacterial activity of a zero-valent silver phase strictly depends on the surface development of the solid, since silver atoms/ions required to accomplish the antibacterial activity are released only from the surface. Consequently, when this solid phase is in a powdered form, the resulting antibacterial activity can be significantly increased, and an ultrafine silver powder may result in several orders of magnitude more activity than the corresponding bulk solid [15]. The Ag NPs are known to oxidize to Ag ions when they interact with water molecules. Ag nanoparticles could provide sustained release of sufficient Ag ion compared to Ag salts and bulk metallic forms due to higher active surface to volume ratios [16]. Finally, the nanosized silver antibacterial activity is enhanced for two main reasons: the fraction of surface silver atoms increases with decreasing particle size, and a greater fraction of surface silver atoms is weakly bonded to the particle surface, which can be easily released to the surrounding medium. It is agreed that the Ag NP nanocomposite antimicrobial activity is basically related to its ability to release Ag^+^ over time [15].

The amount of biotechnological products containing silver nanoparticles in their composition is increasing every day. For example, they are being used in the impregnation of wound dressings, medical devices, dental materials, fabrics, among others, besides the combination with antibiotics for the observation of a synergistic antibacterial effect [13]. Because of the increased attention to antibacterial nanocomposites in the last few decades, there is significant interest in studying Ag ion release from Ag NPs to optimize the nanocomposite performance and to reduce the negative effects on human cells and the environment [17]. Ag nanoparticles have to be placed in media or on a surface, which could have direct contact with skin tissues [18]. As an example, synthetic silk can be rinsed in colloidal silver solution and, later on, dried out. However, in such a case, due to rinsing, less Ag nanoparticles remain, and finally, the surface is polluted with the remainder of the chemicals used in the process [19]. Therefore, this technology is not suitable for bandages. Antibacterial surfaces can be produced, as well as a coating, where Ag-containing coatings can be envisaged as a pure Ag layer, or Ag nanoparticles embedded in a matrix [12].

In this case, diamond-like carbon (DLC) can be considered as a candidate, as it is known as a versatile coating material that finds a variety of biomedical applications, including endoprosthesis and dental implants. It provides mechanical robustness and cell-compatibility at the same time. To broaden this range of beneficial properties even more, trials were reported when silver or silver nanoparticles were embedded in order to add antibacterial properties [4,20].

Considering the final product, it should be mentioned that now, some bandages with silver-coated textile are available on the market [21,22,23], where a thin silver layer is used that may release silver ions if moisture is present. Some manufacturers offer hydrogel-based wound dressings [24,25], where the hydrogel material is saturated with silver ions. In addition, hydrogel contains water and crosslinked molecules, the structure of which ensures moisture absorption [26].

In the current work, DLC:Ag nanocomposite films were deposited on textile (synthetic silk) as a part of a smart bandage prototype demonstrating antibacterial properties. The antibacterial properties of such nanocomposites were systematically tested against *S. aureus* emphasizing conditions of DC magnetron sputtering and, further, plasma processing steps. DLC:Ag properties were characterized employing scanning electron microscopy (SEM) with energy dispersive X-ray spectroscopy (EDS) and atomic absorption spectroscopy (AAS). Further development of the smart bandage by integrating new functionalities is in progress and will be published in the forthcoming publications.

## 2. Results

### 2.1. Structure and Composition of DLC:Ag Layers Deposited on Textile and Crystalline Silicon

From the EDS measurements, it was obtained that the chemical content of Ag in as-deposited DLC:Ag films varies from 0.46–6.43 at % (Table 1).

**Figure 1:**
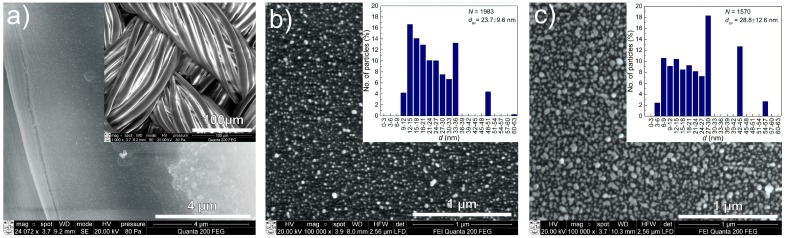
SEM micrographs of DLC:Ag films of different Ag content on different substrates: (**a**) lowest Ag content film (Sample 1; scale bar: 4 µm) on silk; the inset depicts the silk structure (scale bar: 100 µm); (**b**,**c**) summarize the information about the medium and highest Ag concentration film (Samples 2 and 3, respectively; scale bar: 1 µm) on silicon substrates; insets depict the particle size distribution number of analyzed particles (*N*) and the average particle diameters (*d*_av_) of the corresponding SEM micrographs.

SEM measurements revealed that the lowest Ag content films do not have visible Ag nanoparticles on the surface (Sample 1). A few particles were visible only on the surface of the silk substrates (see Figure 1a) that were not present prior to deposition. The middle and highest investigated Ag concentration samples (Samples 2 and 3) had a number of silver clusters on the surface (Figure 1b,c). SEM micrographs and NP analysis are depicted in Figure 1.

### 2.2. Antimicrobial Activity of Virgin and RF O_2_ Plasma Processed DLC:Ag Films

**Figure 2:**
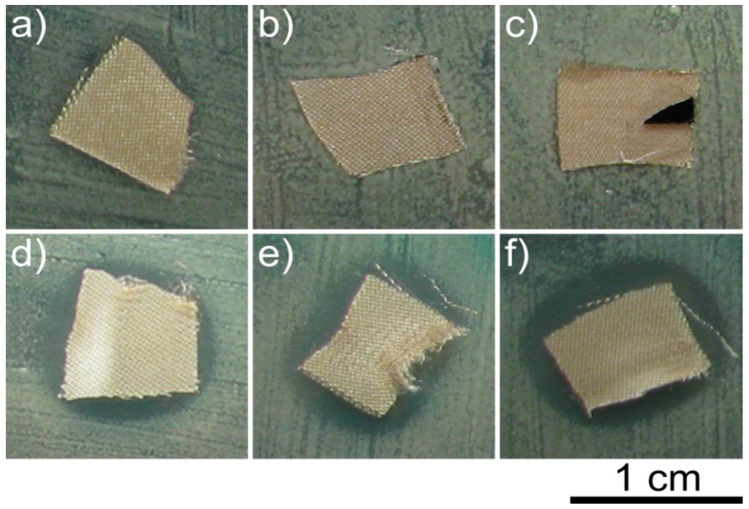
Testing of antimicrobial properties with the *S. aureus* LTSaM01 strain bacteria (disk diffusion method) of virgin Samples 1 (**a**); 2 (**b**) and 3 (**c**); as well as after 20-s plasma processing ((**d**–**f**) respectively). The clear zone in (**d**–**f**) indicates areas where no bacteria multiplication is observed.

Three as-deposited and O_2_ plasma-processed DLC:Ag thin films containing different amounts of Ag 0.5–6.0 at % deposited on synthetic silk were investigated employing the disk diffusion method. Figure 2 demonstrates the results of antimicrobial activity for Samples 1–3.

One can see that the virgin DLC:Ag-coated sample does not show an expressed bacteria inhibition area for the *S. aureus* LTSaM01 strain (the same results were found for all investigated strains around the samples in the Petri dishes), while the O_2_ plasma-processed sample demonstrates up to a 2.5-mm inhibition zone.

**Figure 3:**
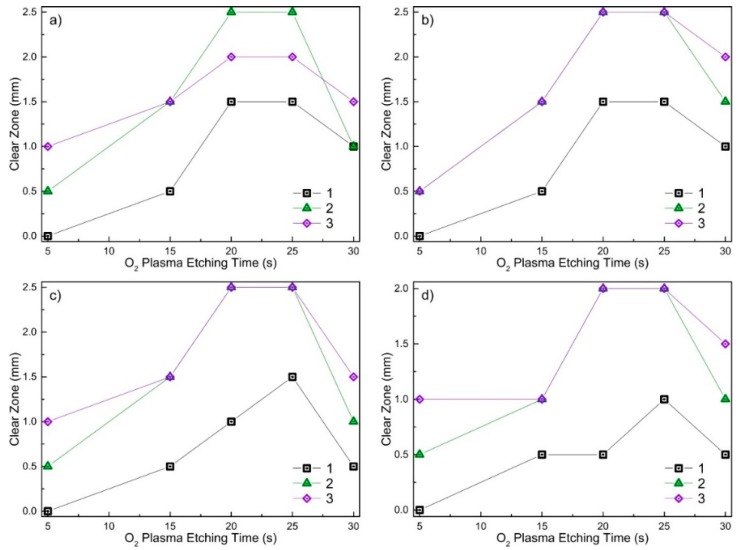
Antimicrobial effect (linear dimensions of the clear zone) for three different Ag concentration DLC:Ag films deposited on silicon and processed with O_2_ plasma for different durations (5–30 s) (Samples 1–3) were tested with four different *S. aureus* bacteria strains, (**a**) LTSaDA01; (**b**) LTSaM01; (**c**) LTSa635 and (**d**) LTSa603, the employing disk diffusion method. Measurement uncertainty: 0.5 mm.

The inhibition zones’ dimensions measured with four different strains after different processing duration with O_2_ plasma for three different Ag concentration samples on silk are depicted in Figure 3.

From Figure 3, one can see that after 5 s of O_2_ plasma etching, Sample 1 showed no antimicrobial effect with all investigated bacteria strains. Sample 2 had only a weak effect for all bacteria strains, *i.e.*, the inhibition zone was ≤0.5 mm (see Figure 3). Sample 3 with 6.43 at % of silver has shown even better results with the shortest plasma processing duration, *i.e.*, the inhibition zone was 1 mm, except bacteria LTSaM01 (see Figure 3b), where a clear zone of 0.5 mm was obtained. After 15 s of etching in O_2_ plasma, Sample 1 had a visible antimicrobial effect for all bacteria strains; the inhibition zone was 0.5 mm. Samples 2 and 3 demonstrated a larger inhibition zone: 1.5 mm; except strain LTSa603 (see Figure 3d), where the inhibition zone was 1 mm. The best results in terms of inhibition zone were observed for the samples etched for 20 s and 25 s. As can be seen from Figure 2d–f and Figure 3, plasma etching strongly increased the inhibition effect for all four *S. aureus* strains for all investigated samples with different Ag content nanocomposite DLC:Ag films.

**Figure 4:**
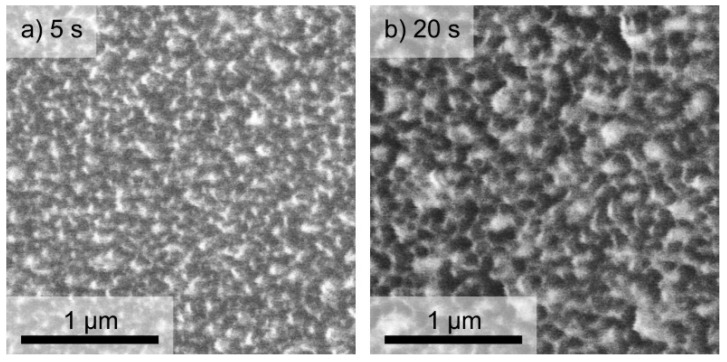
SEM micrographs of DLC:Ag (Sample 2) after different durations of O_2_ processing: (**a**) 5 s; (**b**) 20 s. Scale bar: 1 μm.

**Figure 5:**
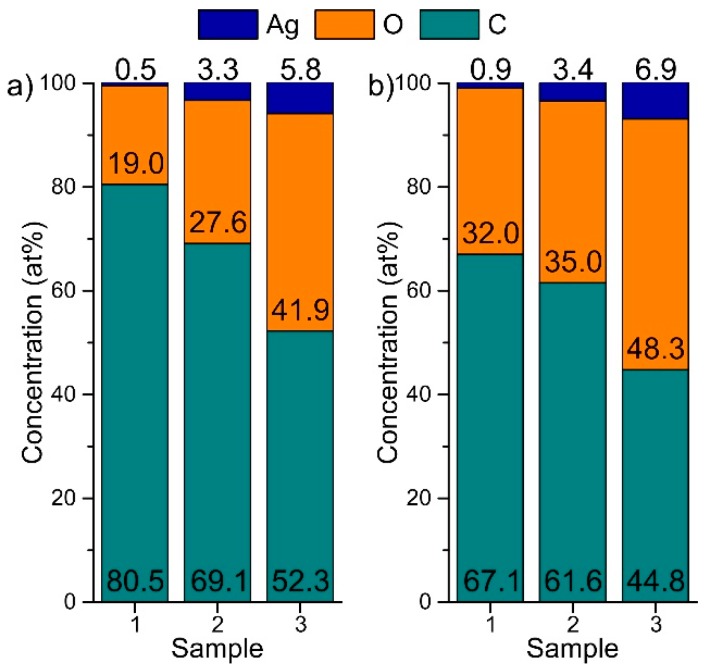
Chemical composition of DLC:Ag films (Samples 1–3) after 5 s (**a**) and 20 s (**b**) of O_2_ plasma processing.

The antimicrobial effect of differently plasma-processed DLC:Ag nanocomposite films on the investigated strains could be related to the surface morphology changes and opening of the Ag NPs’ surface from the DLC matrix (see Figure 4). The chemical composition of the films after different O_2_ plasma processing, providing the characteristic weak and strong antimicrobial effects on the investigated bacteria strains, is depicted in Figure 5.

From Figure 5, one can see that after O_2_ plasma processing, the surface concentration of carbon decreases and the amount of silver increases. Analysis of the composition of the films has shown a relatively high concentration of oxygen in the films as deposited and exposed to oxygen plasma. Unfortunately, these measurements were done after a long exposure of the samples in atmosphere, and potential changes of oxygen concentration in the films were hindered by the surface adsorption processes. Therefore, these values probably could be considered only to identify possible trends of variations but not the absolute (relative) concentration of oxygen.

### 2.3. Antimicrobial Activity of Bandage Prototype

Based on the best antimicrobial activity results, the 20 s plasma-processed DLC:Ag nanocomposite film containing 3.12 at % Ag was selected for further investigation as a building block of the bandage prototype. Synthetic silk substrate was used as a DLC:Ag substrate. A protective layer from cellulose sheet (0.01 mm) gelatin (0.1–0.15 mm) worked as a silver ion accumulation matrix and prevented the bandage from sticking to the wound’s soft tissues. The antimicrobial properties of the prepared prototype were tested using the spread plate technique.

**Figure 6:**
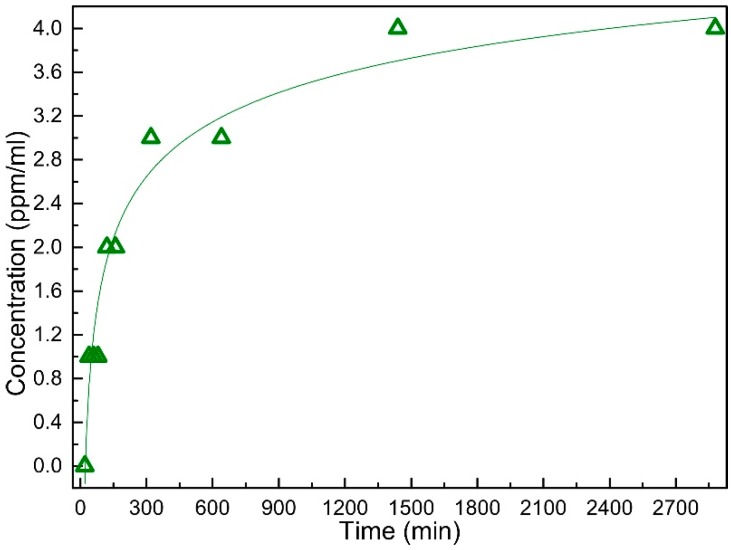
Silver ion concentration in purified water after soaking of synthetic silk coated with DLC:Ag (Sample 2 (3.12 at % Ag)) for different time durations obtained with AAS.

ASS experiments were used to follow the silver ion extraction process from the prototype containing the DLC:Ag nanocomposite layer. Figure 6 depicts silver ion concentration changes for different soaking durations of Sample 2 in purified water.

It was obtained that the silver ion concentration increases sharply during the first 300 min and saturates approximately after 900 min. A double logarithm function (*y* = a × ln(−b × ln(*x*))) can be used to approximate the experimental curve, and a high correlation coefficient *R*^2^ = 0.96 was obtained for the constants a = 4.36 and b = −0.32.

**Figure 7:**
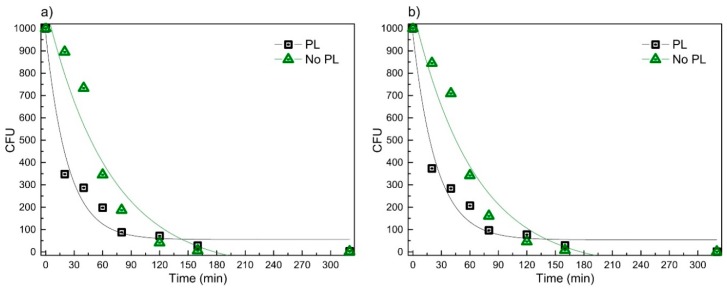

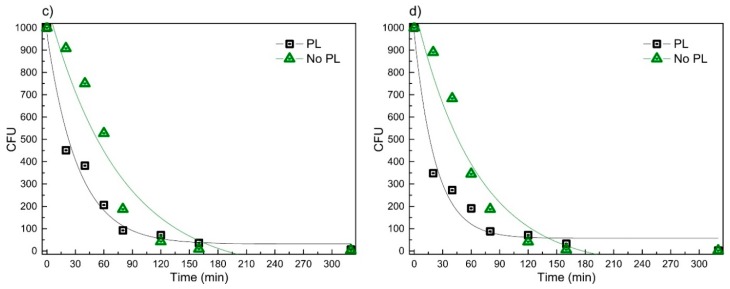
Time dependencies of bacteria colony forming units (CFU) *versus* time using the spread-plate technique for the bandage prototype (PL) and the reference sample (No PL) measured with four types of *S. aureus* bacteria strains: (**a**) LTSaDA01; (**b**) LTSaM01; (**c**) LTSa635 and (**d**) LTSa603.

Antimicrobial testing results of the smart bandage without and with the protective layer obtained using the spread-plate technique with four bacteria strains are summarized in Figure 7.

It was found that the exponential law (equation: *y* = *y*_0_ + *A* × exp(*R*_0_ × *x*) could be used to describe the time dependencies of CFU *versus* time, and Table 2 summarizes the values of the coefficients used in the approximations.

Tests with bacteria LTSaDA01, LTSaM01 and LTSa603 revealed that after 20 min, DLC:Ag-coated synthetic silk without PL killed 10%–15% of *S. aureus* bacteria, when the same material with PL killed 63%–65%. Tests with LTSa635 (MRSA) for the same duration of time have shown efficiencies of 9% and 55%, respectively. Results of the longest soaking duration for 320 min revealed that the bandage without PL had the same antimicrobial effect as compared to the bandage plated with PL, *i.e.*, after bandages, in both cases, 99% of all bacteria were killed, and only a few CFU were observed.

## 3. Discussion

As was presented above, DC-reactive magnetron sputtering appears as an efficient way to produce nanocomposite DLC:Ag coatings. Simple variation of the Ar/C_2_H_2_ flux ratio enables the deposition of films with variable content of silver nanoparticle filler embedded in DLC matrix. The typical structure of the DLC:Ag films deposited under similar conditions can be found in [27]. Similar results were reported, as well, by [28], where DC-reactive unbalanced magnetron sputtering of Ag target in acetylene atmosphere allowed producing DLC:Ag nanocomposite thin films with a variable content of silver. During the experiments, the thickness of the film deposited on crystalline silicon and textile was approximately 40 nm, and according to Figure 1b,c, the average size of silver nanoparticles varied within a range 2–63 nm. In the case of lower silver concentration, low dimension silver nanoparticles prevailed. The average diameters of silver nanoclusters in DLC:Ag nanocomposite films were 23.7 nm and 28.8 nm for Samples 2 and 3, respectively. The larger silver content in the coatings matrix correlated with larger silver cluster diameter. This is in good agreement with our previous experiments, where we have performed deposition of DLC:Ag nanocomposites on silica substrates [29,30].

Antimicrobial results demonstrated that as-deposited DLC:Ag samples with low silver concentration (Samples 1–3) had no or only very weak antimicrobial effect (see Figure 2). As we have defined, additional O_2_ plasma etching appears as an efficient tool in enhancing the antibacterial effect of the DLC:Ag nanocomposite surface. According to the results presented in Figure 5, O_2_ plasma processing reduces the carbon surface concentration and provides the developed nanocomposite surface (Figure 4), which seems close to the results obtained in [31], where O_2_ etching of organic materials provided nanotextured surfaces. It should be noted that plasma etching for 20 s reduced carbon content by 13.9% on average. During this process, a thin layer of carbon from the DLC:Ag surface was removed to expose more silver nanoparticles, which were embedded and covered by the DLC matrix [32]. On the other hand, due to O_2_ plasma bombardment, coalescence of silver particles [33] and partial oxidation of silver nanoparticles take place. From this point of view, Sample 2 appears to be more efficient as compared to Sample 3 in terms of antimicrobial properties due to the smaller size of the nanoparticles. The dominating mechanisms could be elucidated after more comprehensive analysis of the behavior of DLC:Ag, and these experiments are in progress. After the oxygen plasma etching procedure, the average silver content increased by 0.5 at % after 20 s. The reduction of Ag in DLC:Ag films in the case of the highest silver concentration (Sample 3) could be explained by volume expansion and strain-induced cracking of oxidized Ag NPs due to oxygen ion bombardment [34,35]. Probably, a high concentration of silver, as well as an increase of the size of the nanoparticles in the case of Sample 3 (as compared to the other two samples) contribute to the efficiency of the mentioned mechanism. According to [36], the improvement of antimicrobial properties correlates with the increased hydrophobicity and, according to our findings, with the increased surface concentration of silver, as well.

As one can see, the etching time affected structural surface changes (Figure 4). Comparing samples etched for 5 s and as-deposited samples, only minor surface changes were observed, but the samples exposed to O_2_ plasma for the longer time had many small dimples instead of a smooth surface. According to [36], this surface process is defined by the reaction of carbon materials, like DLC films with oxygen plasma, where plasma produces a destruction of the graphite rings. This results in an increase of single carbon chains where the concentration of aliphatic carbons atoms is pre-dominant in O_2_ plasma-treated DLC films. Oxygen plasma treatment makes the DLC surface more desorbed, rougher and superhydrophilic [36]. Finally, the superhydrophilic surface ensured maximum silver ion diffusion from the samples to wet agar media. On the other hand, we have found that the prolonged plasma exposure (above 25 s) resulted in the decrease of antibacterial activity for all investigated strains and investigated silver concentrations (see Figure 3). This effect could be attributed to the ion beam irradiation-induced increase of silver nanoparticles (ripening process) that we have observed early in the case of reactive ion etching of DLC:Ag nanocomposites [33]. One can expect that the larger particles have a smaller surface area, which is responsible for silver ion release into media. The nanoparticle size effect on the antimicrobial properties was reported as well in [37,38], where the authors declare better antibacterial properties of small dimensions of AgNPs.

According to our findings, the optimal time for RF oxygen plasma etching was 20–25 s (see Figure 3). For such plasma exposure, all of the coatings with different silver content revealed the best antimicrobial properties, *i.e.,* they had the developed surface and optimum dimensions of the silver nanoparticles.

For such kinds of samples (Sample 2 as a typical example was taken (silver content after 20 s; etching was 3.41 at %)), AAS analysis revealed efficient silver ion migration into aqueous media (Figure 6). We found out that saturation of silver ion concentration in purified water takes place, and after 24 h, at a 35 °C temperature, it reaches 4 ppm. It should be noted that according to [38], the antibacterial activity against *S. aureus* starts at about 1 ppm. These results correlate well with the microbiological testing (spread-plate technique; Figure 7) data where larger silver concentrations killed more bacteria, as well.

Experimental data (Figure 7) revealed that the bandage without PL (soaking duration of 20 min) demonstrated a lower antimicrobial effect as compared to the bandage where PL was applied. One can assume that our chosen aqueous media (PL) can accumulate silver ions inside. After 24 h of exposure of the bandage, the PL has accumulated silver ions and during the test acted as an efficient source of silver ions. Moreover, the agar-gelatin layer at 35 °C can dissolve easily, and silver ions can spread rapidly to all aqueous media, providing a very good antimicrobial effect. In all studied cases, PL technology improved the antibacterial properties of the bandage, and it was more than approximately 50% effective during the same time interval. The action speed was the main advantage of the prototype, *i.e.,* PL can be used as silver ion accumulation media for fast ion release in aqueous media, enabling to kill instantly more than 50% of all of the bacteria population. It should be noted that in wound healing, this is a very important factor, as the bacteria need to be killed in a short period of time to prevent efficient growth of the bacteria population [39,40,41]; e.g., in [16], it was shown that bacterial adhesion involved reversible bacterial association in the first 1–2 h after post-implantation, followed by stronger bacterial adhesion, approximately 2–3 h later. After 24 h, certain bacteria formed a biofilm, which was resistant to host defense and systemic antibiotic treatment.

In addition to the efficient antimicrobial properties, our bandage with PL having a gel-like structure with the synthetic silk skeleton ensures good mechanical properties, as well. The cellulose sheet as the membrane can sustain small particles and debris inside the bandage close to the DLC:Ag surface, avoiding wound contamination. The gelatin and agar layer has very good water absorption abilities, as was demonstrated in [42]. Furthermore, according to [14], approximately 39% more silver is released into alkaline sweat (pH 8.0) as compared to acidic sweat (pH 5.5). In infected wound, pH usually moves to a neutral or slightly alkali pH value; in that case, our technology also has an advantage: silver ions should migrate faster, from nanoparticles inside the DLC matrix, and the antimicrobial effect could be even stronger comparing to the tests in saline solution (0.9%) [14]. The higher release rate of Ag ion concentration into the surrounding medium and the longer it is sustained, the more thorough the antimicrobial effect will be [43].

It should be noted as well that cytotoxicity is one of the main problems with using nanotechnology in medical devices like bandages. Embedded cells like nanoparticles can cause adverse side effects to the organism. In our bandage prototype, only the PL structure was used, to avoid this problem, but further tests are needed to investigate toxicity. In the prototype bandage, the silver ion concentration, according to the AAS data, could reach 4 ppm or 4 μg/mL. According to [13], the minimum inhibitory concentrations of all bacteria tested were in a concentration range of AgNPs (between 3.37 and 13.5 μg/mL) in which there was no observed significant cytotoxic activity compared to the control [13].

## 4. Materials and Methods

### 4.1. Deposition, Characterization and O_2_ Plasma Processing of DLC:Ag Films

DLC:Ag coatings with different Ag content were deposited on textile (twill weaved synthetic silk, with a weft density of 110 cm^−1^ and a warp density of 100 cm^−1^) by DC-reactive unbalanced magnetron sputtering of Ag target in acetylene atmosphere. Ag content was controlled varying feed stock gas flow rates and changing magnetron power, as well as bias voltage. Crystalline silicon wafer substrates were used as well for the comparative control of the deposition process. The deposition conditions are summarized in Table 3. Further details on the deposition and properties of DLC:Ag coatings with variable silver concentration can be found in our early papers [33,44,45].

The thickness of the deposited DLC:Ag coatings was approximately 40 nm (as measured by scanning electron microscope (SEM)). The AgNPs’ particle size distribution and chemical composition were obtained employing SEM FEI Quanta 200 FEG with an energy dispersive X-ray spectrometer (EDS), Bruker Quantax. NPs’ diameters and the chemical composition of the films were studied for the DLC:Ag samples deposited on Si substrates. EDS measurements were performed at a5-keV accelerating voltage in order to minimize the excitation of the Si K_α_ peak. NP size analysis was preformed employing ImageJ (NIH) software and custom MATLAB (MathWorks) code. More detailed information about the applied NP analysis procedures can be found elsewhere [33,46]. Measurements of as-deposited nanocomposite thin film were performed at 3–5 different points, and average normalized surface concentrations (excluding silicon) can be found in Table 1.

After the deposition, the samples were additionally etched by radio frequency (RF; 13.56 MHz) oxygen plasma (99.9%) in 133 Pa pressure and 0.3 W/cm² power for 5–30 s. Morphology and antimicrobial properties of the as-deposited and O_2_ plasma-etched DLC:Ag films on silicon and silk were investigated.

### 4.2. Microbiological Testing of Virgin and O_2_ Plasma-Processed DLC:Ag Films

**Figure 8:**
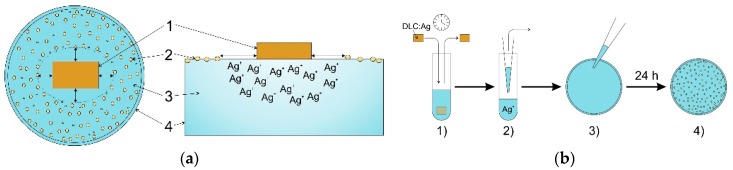
Antimicrobial testing techniques. (**a**) Schematics of the disk diffusion method used for microbiological testing (Ag^+^ diffusion of silver ions from the DLC:Ag surface into agar): (**1**) sample coated with DLC:Ag nanocomposite; (**2**) bacteria colonies; (**3**) agar (bacteria nutrition media); (**4**) Petri dish; (**b**) Schematics of the spread plate technique: (**1**) bandage prototype soaked in a test tube with bacteria saline solution for different time durations; (**2**) the bacterial solution with Ag^+^ ions (0.1 mL) was transported into a Petri dish; (**3**) inoculation of bacteria onto the agar surface; (**4**) calculation of colony forming units (CFU).

For testing of the bactericidal activity of DLC:Ag films on multiplying bacteria, four clinically-important strains causing skin and wound infections of *S. aureus* previously isolated at Lithuanian University of Health Sciences were selected. Two strains were isolated from sick humans (LTSaDA01 and LTSaM01), as well as two strains were isolated from diseased pet animals: a dog (LTSa603) and a cat (LTSa635). The strain LTSa635 was methicillin-resistant. The antimicrobial properties of the DLC:Ag layer, as well as bandage prototype including the DLC:Ag layer were tested using the disk diffusion method (see Figure 8a) and the spread-plate technique [47] (see Figure 8b). The bacterial suspension density of 1 McFarland unit in saline solution (0.9%) was prepared and inoculated onto Mueller Hinton agar (Thermo Scientific, Leicestershire, UK) in 94 mm-diameter Petri dishes. In the disk diffusion method, synthetic silk samples (virgin and etched) of a 6 mm × 6 mm (±1 mm) size with DLC:Ag coatings (Samples 1–3) were glued onto the agar surface using a small drop of water. The samples were incubated for 24 h at 35 °C. The dimensions of inhibition zones were measured from four sides of the sample, and the average value of the clear zone was calculated. To check the temporal stability of the coatings, experiments with all three different silver concentration DLC:Ag samples on synthetic silk were repeated for 4 times during a one-month period: at first day and thereafter at the 1st, 2nd and 3rd week after the deposition. Experimental results were fitted employing OriginPro (OriginLab, Northampton, MA, USA).

### 4.3. Construction of the Bandage Prototype

**Figure 9:**
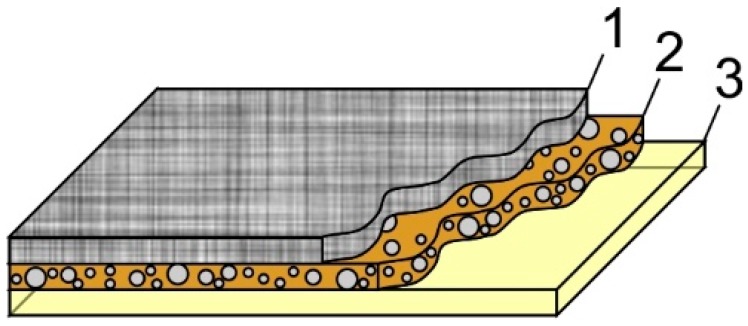
Principle structure of the proposed bandage: (**1**) synthetic silk substrate; (**2**) thin nanocomposite DLC:Ag films etched for 20 s in O_2_ plasma; (**3**) protective layer made from the thin cellulose sheet (membrane) and the gelatin layer.

DLC:Ag coating on synthetic silk indicated the best antimicrobial properties and was used as a part of the smart bandage. The coating was exposed to UV irradiation for disinfection. As a protective layer (PL), cellulose fibers (medium, C6288 Sigma, Sigma Aldrich, St. Louis, MO, USA), gelatin (53028 FLUKA, Sigma Aldrich) and agar (A1296 SIGMA, Sigma Aldrich) were used. A thin cellulose sheet, acting as a membrane, was manufactured from microfiber cellulose and gelatin as a gluing material. It was rolled into a 0.01 mm-thick sheet. The cellulose sheet was glued using gelatin and pressed using a metal roller on DLC:Ag-coated synthetic silk. A hot suspension from gelatin and agar (90%/10%, respectively) was prepared and placed on top as a second layer using a spin coater at low speed (120 rpm). The structure of the smart bandage is presented in Figure 9. In such way, the prepared bandage prototype was used in the antibacterial tests after 24 h.

### 4.4. Antimicrobial Testing of the Bandage Prototype

Bactericidal activity evaluation tests of the bandage prototype were carried out using the same four strains of *S. aureus* as described Section 4.2. The spread-plate technique [47] was applied to confirm the bandage’s ability to kill bacteria on the surface and in the liquid media around it. The protective layer-covered rectangular-shaped bandage of 100 mm^2^ in area was soaked for 20, 40, 60, 80, 120, 160, 320 min in a thermostat at 35 °C in a 1-mL volume of bacteria in saline solution of 0.1 McFarland units. To check the efficiency of the protective layer, an identical experiment was carried out with the bandage including just the nanocomposite layer (Sample 2 etched for 20 s). After incubation, the withdrawn solution was diluted 1:1000 times, and thereafter, 100 µL of bacteria suspension were inoculated onto a Petri dish containing Mueller Hinton. After 24 h, colony forming units (CFU) were counted. For statistical reliability, this experiment was repeated 3 times.

### 4.5. Antimicrobial Testing of the Bandage Prototype

Atomic absorption spectroscopy (AAS) was used to identify Ag^+^ migration from the bandage (synthetic silk with Ag nanoparticles) to ultrapure water. The disk-shaped 10-cm^2^ bandage was soaked in the test tube with 10 mL of thermostated water at 35 °C for time intervals from 20 min to 48 h. Later on, water was filtrated from large (0.1 mm) particles, and AAS measurements were performed. A Perkin Elmer Model 403 spectrometer was employed.

## 5. Conclusions

Diamond-like carbon-based silver nanocomposite layers deposited by DC-reactive magnetron sputtering on textile appeared as an effective source of Ag^+^ ions and demonstrated expressed antibacterial properties against four tested strains of *Staphylococcus aureus* bacteria.

The efficiency of the Ag^+^ ion release to the aqueous media can be increased by further RF oxygen plasma etching of the nanocomposite. It was obtained that the best antibacterial properties were demonstrated by the plasma-processed DLC:Ag layer having a 3.12 at % Ag surface concentration with the dominating linear dimensions of nanoparticles being 23.7 nm.

An extra protective layer made from cellulose and gelatin with agar contributed to the accumulation and efficient release of silver ions to the aqueous media, increasing the bandage antimicrobial efficiency up to 50% as compared to the single DLC:Ag layer on textile.

The proposed bandage prototype (having a silver ion concentration in the protective layer below the toxic level for organism cells) was able to kill more than 99.9% of all strains of bacteria after 320 min, including methicillin-resistant *Staphylococcus aureus*.

## Figures and Tables

**Table 1:** Averaged DLC:Ag chemical composition obtained with EDS.

Sample No.	Carbon (at %)	Silver (at %)	Oxygen (at %)
1	67.54 ± 1.33 ^1^	0.46 ± 0.02	32.01 ± 1.33
2	61.55 ± 0.58	3.12 ± 0.11	31.16 ± 4.65
3	48.92 ± 3.65	6.43 ± 0.65	44.65 ± 4.27

^1^ Uncertainty is one standard deviation.

**Table 2:** Coefficients of exponential dependencies used in the approximations of CFU *versus* time for four types of *S. aureus* bacteria strains.

Sample Structure	Fitting Coefficients	LTSaDA01 (a)		LTSaM01 (b)		LTSa635 (c)		LTSa603 (d)	
		Value	S.E. ^1^	Value	S.E.	Value	S.E.	Value	S.E.
PL	*y*_0_	55.37	36.36	54.13	34.42	30.77	35.16	56.566	33.60
	*A*	921.2	74.29	922.4	68.84	943.4	61.89	922.87	69.53
	*R*_0_	−0.0430	0.00826	−0.0407	0.00717	−0.0305	0.00463	−0.0445	0.00803
	*R*^2^	0.956		0.962		0.971		0.961	
No PL	*y*_0_	−83.45	110.3	−75.39	97.95	−101.1	130.6	−77.21	95.29
	*A*	1184.3	134.41	1157.7	121.45	1208.68	149.92	1170.6	117.74
	*R*_0_	−0.0149	0.00408	−0.0154	0.0039	−0.0132	0.00397	−0.0153	0.00371
	*R*^2^	0.919		0.930		0.908		0.936	

^1^ Standard Error.

**Table 3:** Deposition conditions of DLC:Ag films.

Sample No.	Sputtering Duration (s)	Ar Gas Flow (sccm)	C_2_H_2_ Gas Flow (sccm)	Magnetron Voltage (V)	Magnetron Current (A)
1	520	70	21.1	553–625	0.07–0.12
2	235	70	21.1	568–741	0.07–0.22
3	200	80	7.8	625–656	0.10–0.11

## Abbreviations

The following abbreviations are used in this manuscript

DLC	diamond-like carbon
DLC:Ag	diamond-like carbon with silver nanoparticles
RF	radio frequency
MRSA	methicillin-resistant *Staphylococcus aureus*
*S. aureus*	*Staphylococcus aureus*
Ag^+^	silver ions
DNA	deoxyribonucleic acid
Ag NPs	silver nanoparticles
DC	direct current
PL	protective layer
SEM	scanning electron microscopy
EDS	energy dispersive X-ray spectroscopy
AAS	atomic absorption spectroscopy
*N*	number of analyzed particles
*d*_av_	average particle diameter
*a*	constant
*b*	constant
*R*^2^	correlation coefficient
y_0_	coefficient of extrapolation
*A*	coefficient of extrapolation
*R*_0_	coefficient of extrapolation
S.E.	standard error
